# drGT: Attention-Guided Gene Assessment of Drug Response Utilizing a Drug-Cell-Gene Heterogeneous Network

**Published:** 2025-08-27

**Authors:** Yoshitaka Inoue, Hunmin Lee, Tianfan Fu, Augustin Luna

**Affiliations:** 1 Computer Science, University of Minnesota, 200 Union Street SE, 55455, MN, US; 2 Computational Biology Branch, National Library of Medicine, 8600 Rockville Pike, 20894, MD, US; 3 Developmental Therapeutics Branch, National Cancer Institute, 9000 Rockville Pike, 20892, MD, US; 4 Computer Science, Rensselaer Polytechnic Institute, 110 8th Street, 12180, NY, US

**Keywords:** Drug Response Prediction, Graph Neural Networks, Heterogeneous Networks, Interpretability

## Abstract

A challenge in drug response prediction is result interpretation compared to established knowledge. drGT is a graph deep learning model that predicts sensitivity and aids in biomarker identification using attention coefficients (ACs). drGT leverages a heterogeneous graph composed of relationships drawn from drugs, genes, and cell line responses. The model is trained and evaluated using major benchmark datasets: Sanger GDSC, NCI60, and Broad CTRP, which cover a wide range of drugs and cancer cell lines. drGT demonstrates AUROC of up to 94.5% under random splitting, 84.4% for unseen drugs, and 70.6% for unseen cell lines, comparable to existing benchmark methods while also providing interpretability. Regarding interpretability, we review drug-gene co-occurrences by text-mining PubMed abstracts for high-coefficient genes mentioning particular drugs. Across 976 drugs from NCI60 with known drug-target interactions (DTIs), model predictions utilized both known DTIs (36.9%) as well as additional predictive associations, many supported by literature. In addition, we compare the drug-gene associations identified by drGT with those from an established DTI prediction model and find that 63.67% are supported by either PubMed literature or predictions from the DTI model. Further, we describe the utilization of ACs to identify affected biological processes by each drug via enrichment analyses, thereby enhancing biological interpretability. Code is available at https://github.com/sciluna/drGT.

## Introduction

Drug discovery is an expensive and lengthy process with many obstacles [28]. The difficulty of this process arises from the efforts needed to ensure a compound is both safe and effective. Sensitivity to a drug involves the mechanism of the drug compound and a complex interplay of various factors internal and external to a cell. These factors include the cellular context (or state) that is determined by the repertoire of transcripts and proteins, alterations to this repertoire due to disease, and the interactions between these components [8]. Biomarkers are essential for understanding the use of drug compounds and aiding our understanding of disease biology [10]. Machine learning (ML) has emerged as an approach to understanding the role of particular genes in drug response [30]. When it comes to understanding biological phenomena, ML algorithms, especially deep learning (DL) models, have demonstrated their capability to predict complex patterns and relationships within biological data [40].

However, despite advances in using ML for drug discovery [37], a critical difficulty remains; the “black box” nature of the methods. While DL models have made significant strides in identifying patterns and predicting outcomes across various areas, their internal decision-making process remains unclear, raising concerns about their trustworthiness and reliability [13]. Despite recent efforts to improve interpretability, ML models can fail to yield meaningful insights in biological contexts. Many models in biomedicine offer strong predictive performance without describing the underlying biological associations leading to predictions, thereby limiting their utility for scientific discovery and clinical decision-making [41]. In these areas, performance metrics alone are inadequate; models must meet standards of biological plausibility to function suitably for hypothesis generation [9]. These limitations highlight the need for models that offer both accurate predictions and interpretable representations of biological systems. Interpretability in ML has received significant recent consideration [26], including in the area of drug discovery [21]. The attention mechanism, introduced in the Transformer architecture [55], is used due to its capacity to include “attention coefficients” (ACs) as trainable parameters to optimize how much attention the model assigns to individual components, such as a word in a sentence (*i.e.*, a linear sequence of data); these attention parameters can be used to interpret the model’s output. While words in a sentence express a single coherent idea, regulatory network biology is more dependent on interconnections between components; this makes graph data structures an appropriate representation in biology. Graph Neural Networks (GNNs) have been developed for handling this type of data. GNNs aggregate node-level features in local graph neighborhoods, thereby forming features that also incorporate node relations. GNNs that utilize the attention mechanism are known as Graph Transformer (GT) [49]. Thus, GT generates ACs that weigh the influence of feature representations from neighboring nodes.

We propose an interpretable deep learning method called ‘drGT’, which leverages GT to process a large-scale heterogeneous network. Our model processes a heterogeneous graph composed of drug compounds, cell lines, and genes. This heterogeneous graph has been constructed using integrated drug screening and molecular profiling data from the NCI60 [50], Genomics of Drug Sensitivity in Cancer (GDSC) [61], and Cancer Therapeutics Response Portal (CTRP) [46] datasets, taken from CellMinerCDB [38] via rcellminer [39], and also a drug-target interaction (DTI) dataset from DrugBank [58]. The GT layer is employed to learn embedding representations from the interconnected structure within this heterogeneous network. This approach gives us knowledge of the relative significance of individual components [62] and their contributions to the overall model. We utilize drGT for two tasks: i) drug-cell line association prediction and ii) interpretability of the individual gene importance to prediction. To evaluate performance, we conducted two tests: (1) random masking of the response matrix with reconstruction to assess the model’s imputation accuracy in recovering missing drug-response values, and (2) leave-one-out generalization, withholding all data for a cell line or drug to evaluate the model’s ability to generalize and predict unseen cell lines and drugs. drGT achieves state-of-the-art (SOTA) or comparable performance while also providing interpretability by leveraging ACs, which highlight the relative importance of different nodes or features in the prediction process. We present an ACs analysis for gene nodes that propose explanations for drug responses. In contrast to existing interpretable methods (*e.g.*, SubCDR [36]; subcomponent-guided DL), which rely on coarse-grained molecular fragments and gene subsets for interpretability, drGT provides finer-grained attributions at the drug-gene interaction level by leveraging ACs assigned to drug-gene edges in the graph. This edge-level interpretability enables us to quantify the contribution of each gene to a given drug’s response, yielding biologically detailed and actionable insights. To validate the biological plausibility of these associations, we benchmark the AC-derived drug-gene links against PubMed literature co-occurrence and predictions from DeepPurpose [24], a state-of-the-art DTI model. This validation strategy helps in the understanding of how our resulting drug-gene associations are biologically grounded as well as computationally supported. Moreover, relevant biological processes are described using the ACs and an over-representation analysis. This work makes three main contributions. First, we create a heterogeneous graph including three entity types: genes (*i.e.*, gene expression), cell lines (*i.e.*, drug responses), and drugs (*i.e.*, structures) using multiple datasets (*e.g.*, NCI60, GDSC, and CTRP). Second, we evaluate the drug-gene associations (DGAs) suggested by ACs and compare these to existing biomedical literature. We examine the abstracts of journal papers for co-mentions of drug-target relationships from our results. Note that while DTIs refer to a direct physical or biochemical interaction, DGAs may or may not imply such direct binding. Additionally, we note that for the purposes of this study, we define interpretability as the ability to reveal biologically meaningful factors contributing to drug response, such as key genes or subcomponent interactions, via mechanisms like attention weights or interaction scores. Third, drGT achieves AUROC scores up to 94.5% on benchmark datasets while maintaining interpretability. drGT enables biologically meaningful analysis of gene importance and pathway enrichment through ACs.

## Methods

### Building Input Matrix

#### Preprocessing

First, we selected a subset of data from NCI60 [50], GDSC1, GDSC2 [61] and CTRP [46] via rcellminer [39]. This dataset comprises gene expression and drug structure data. Drug response data were obtained as IC50s from the PharmacoDB database [51]. The drug response matrix contained 23,191 drugs for 60 cell lines in the NCI60 dataset, 339 drugs for 987 cell lines in the GDSC1 dataset, 188 drugs for 987 cell lines in the GDSC2 dataset, and 496 drugs for 1,036 cell lines in the CTRP dataset. However, due to missing expression data for one cell line in the NCI60 gene expression dataset (*i.e.*, SF539), only 59 cell lines were ultimately used for downstream analyses. From this, we selected 976 drugs (See Table 1) with various mechanisms of action (MoAs) (*e.g.*, DNA damage, tubulin, kinase, apoptosis, HDAC, HSP90, proteasome, bromodomain (BRD) inhibition) and drugs overlapping with other datasets (GDSC and CTRP) for NCI60 to be feasible on an NVIDIA A100 GPU (80GB). Specifically, the drug response matrix forms $X_{DR}\in {\mathbb{R}}^{n\times m}$, where n is the number of drugs and m is the number of cell lines (*i.e.*, n=976, m=59). For CTRP/GDSC dataset, we included all compounds, along with their gene expression and drug response data. After compound filtering, IC50 values were log10-transformed and filtered to the central 95% range (2.597.5 percentiles). After Z-score normalization per drug, values were binarized using a margin-based threshold:

$$\text{Label}=\left\{\begin{array}{lll} 1 & \text{if}\;x<\mu -\sigma & \text{(Sensitive)}\\ 0 & \text{if}\;x>\mu +\sigma & \text{(Resistant)}\\ \text{NaN} & \text{otherwise} & \text{(Uncertain)} \end{array}\right.$$

where μ and σ denote the mean and standard deviation of IC50 values for each drug. Pairs labeled “NaN” were excluded from training and evaluation.

The NCI60 gene expression data includes 23,059 gene transcripts for 59 cell lines, where we selected 2,247 genes (the top 10% of the genes with the highest standard deviation). We also merged another group of genes (242 genes) that are involved in DTIs. This combination resulted in a unique set of 2,489 genes. The gene expression data is part of matrix $X_{GE}\in {\mathbb{R}}^{m\times l}$, where m=59 and l=2,489 denote the number of cell lines and genes, respectively. Similarly, the GDSC1 dataset contains gene expression data for 925 cell lines across 2,106 genes; GDSC2 for 654 cell lines across 2,040 genes; and CTRP for 807 cell lines across 2,160 genes.

Subsequently, we collected DTI data from DrugBank [58], comprising 19,017 interactions between 7,756 unique drugs and 4,755 target proteins. We selected 191 unique National Service Center (NSC) numbers (NSCs represent batches of screening with a particular drug structure) and 242 genes, with drugs and genes overlapping with the NCI60 pharmacogenomic data. The matrix $X_{DTI}$ is zero-padded or filled with 0.5 for unknown DTI to match dimensions, where $X_{DTI}\in {\mathbb{R}}^{n\times l}$ (n=976, l=2,489); the choice between zero-padding and 0.5 is treated as a hyperparameter. Similarly, drug-target subsets were selected for GDSC1 (82 drugs, 178 genes), GDSC2 (49 drugs, 100 genes), and CTRP (110 drugs, 214 genes) (See Table 1).

Additionally, we created a drug fingerprint matrix. SMILES structures are converted to the Morgan fingerprints [42] using RDKit [29]. We create a vector of 2048 length for each drug and concatenate to make a matrix, denoted as $X_{MF}\in {\mathbb{R}}^{n\times o}$, wherein o represents the number of drug features (*i.e.*, n=976, o=2,048). Similarly, corresponding fingerprint matrices were constructed for the GDSC1, GDSC2, and CTRP.

#### Feature Matrix

To make a feature matrix, we utilize similarity matrices as inputs created from $X_{GE}$, and $X_{MF}$. Similarity matrices were constructed for cell lines $\left(S_{c}\right)$, genes $\left(S_{g}\right)$, and drugs $\left(S_{d}\right)$. These matrices capture the element-wise similarity to homogenous entities such as drug-drug similarity. The RBF kernel was used to create similarity matrices where $S_{ij}=\exp\left(-\gamma {\left\|X_{i}-X_{j}\right\|}^{2}\right)$. Here, S represents the similarity matrix, γ is calculated as 1/length $\left(X_{i}\right)$, and X is the input matrix. This equation was applied to $X_{GE}$, $X_{GE}^{T}$, and $X_{MF}$ to obtain cell $\left(S_{c}\right)$, gene $\left(S_{g}\right)$, and drug $\left(S_{d}\right)$ similarity matrices, respectively. As a result, three similarity matrices were obtained: $S_{c}\in {\mathbb{R}}^{m\times m}$ for cell lines, $S_{d}\in {\mathbb{R}}^{n\times n}$ for drugs, and $S_{g}\in {\mathbb{R}}^{l\times l}$ for genes. These matrices have different dimensions and we utilize three linear layers to transform them to the same size of hidden units. The unified matrix X is defined as follows:

(1) $$X=\left[\begin{array}{ccc} {\text{Linear}}_{d}\left(S_{d}\right) & 0 & 0\\ 0 & {\text{Linear}}_{c}\left(S_{c}\right) & 0\\ 0 & 0 & {\text{Linear}}_{g}\left(S_{g}\right) \end{array}\right],$$

where each linear layer has a different input size and the same output size. The output size is a hyperparameter.

#### Adjacency Matrix

We also create the adjacency matrix from $X_{DR}$, $X_{GE}$, and $X_{DTI}$. The drug-cell association matrix $A_{dc}$ is derived from the drug response matrix $X_{DR}\in {\mathbb{R}}^{n\times m}$, where each entry is either 0 or 1. Based on this matrix, we constructed $A_{dc}$. Subsequently, we establish a cell-gene association matrix, $A_{cg}$, derived from the gene expression dataset $X_{GE}\in {\mathbb{R}}^{m\times l}$. Our initial step includes standardizing the data across columns (cell lines). Then, the cell-gene association matrix $A_{cg}$ is generated from $X_{GE}$ by retaining only positive values. Non-positive entries are set to zero, resulting in a sparse matrix that highlights genes with above-average expression in each cell line. Lastly, we create the association matrix between drugs and genes from the matrices $X_{DTI}$. The matrix is initialized with either 0 or 0.5 to represent unknown interactions, depending on a hyperparameter setting. Then, known drug-gene interactions are assigned a value of 1. The resulting drug-gene association matrix $A_{dg}\in {\mathbb{R}}^{n\times l}$ retains all known interactions, while unknown entries remain at their initialized values.

Based on the three matrices, a unified adjacency matrix A is created in Equation (2) as follows

(2) $$A=\left[\begin{array}{ccc} 0 & A_{dc} & A_{dg}\\ A_{dc}^{⊤} & 0 & A_{cg}\\ A_{dg}^{⊤} & A_{cg}^{⊤} & 0 \end{array}\right].$$

$A\in {\mathbb{R}}^{\left(n+m+l)\times (n+m+l\right)}$ represents the combined drug, cell, and gene components. The input matrix X and the adjacency matrix A are utilized for the input of the GNN model.

To prevent data leakage, we masked the test data in the drug-cell association matrix by setting their values to 0.

### drGT Model

This model consists of GNN layers and fully connected (FC) layers. We utilized Graph Transformer (GT) [49] as the GNN layer. GT introduces a dynamic attention mechanism that allows each node to attend to others, improving flexibility. By integrating multi-head attention, GT aggregates information from diverse neighborhoods. Equation (3) describes the first block of the GT layer with feature matrix X and adjacency matrix A. The number of GNN and FC layers is treated as a hyperparameter (Appendix A) and may vary depending on the model configuration. The subsequent steps involve adapting the model’s output to predict drug sensitivity in a binary manner.

(3) $$Z_{1}=\mathrm{Dropout}(\mathrm{ReLU}(\mathrm{GraphNorm}(\mathrm{GT}(X,A)))),$$

where Dropout denotes the dropout layer, ReLU is a ReLU activation function, GraphNorm is a graph normalization layer [7], and GT is a Graph Transformer Layer. The following GNN layer utilizes equation (3) with $Z_{1}$ as the input and we obtained $Z_{2}$. Drug-cell line associations are predicted by concatenating their respective embeddings, and passing this input through FC layers followed by a sigmoid activation.

Equation (4) details the entire computational procedure. Then, the predicted value, $\hat{y}$ is obtained by taking the sigmoid function to the output of FC layers as follows:

(4) $$\hat{y}=\mathrm{sigmoid}\left(\mathrm{FC}\left(Z_{2d}\|Z_{2c}\right)\right),$$

where the $Z_{2d}$ and $Z_{2c}$ are referred to as the drug’s and cell’s embedding from the $Z_{2}$, respectively, and ‖ describes concatenation. The predicted output $\hat{y}$ and ground truth y are fed into the binary cross entropy loss $L:L(y,\hat{y})=-\frac{1}{N}\sum_{i=1}^{N}\left[y_{i}\log\left({\hat{y}}_{i}\right)+\left(1-y_{i}\right)\log\left(1-{\hat{y}}_{i}\right)\right]$, where N is the total number of training samples. The network is optimized by Adam [27]. Our model was implemented with PyTorch 0.13.1 [43] and PyTorch Geometric 2.2.0 [20]. The hyperparameters were tuned with Optuna [2]. The search space included hidden sizes, dropout, GNN/MLP layers, attention heads, activation functions, optimizers, normalization methods, learning rate, weight decay, and epochs. Details are in Appendix A.

## Results

### Evaluation Protocol

We evaluated our model on four benchmark datasets—GDSC1, GDSC2, CTRP, and NCI60—using two metrics: area under the receiver operating characteristic curve (AUROC) and area under the precision-recall curve (AUPR). Following the protocol from the Neighborhood Interaction-based Heterogeneous Graph Convolutional Network (NIHGCN) [45], we implemented two evaluation settings:

#### Imputation of Randomly Masked Values with 5-Fold Cross Validation (Test 1):

Using 5-fold cross validation, 20% of drug-cell line associations were randomly masked to assess the model’s imputation capability through reconstruction of masked entries.

#### Performance to Unseen Drugs or Cell Lines using Leave-One-Out Evaluation (Test 2):

All associations for a drug or cell line were masked to examine the model’s ability to generalize to novel drugs or cell lines without prior interaction data, thus simulating a cold start setting.

To prevent information leakage, all methods applied a binary mask M to the drug-cell adjacency matrix $A_{dc}$, where $M_{ij}=0$ for test entries. Training used the element-wise product $A_{dc}⊙M$, where ⊙ denotes the Hadamard product.

### Comparison Models and Baseline Architectures

To contextualize the performance of drGT, we compared it against five SOTA models: DeepDSC [32], Multi-Omics Fusion and Graph Convolution Networks (MOFGCN) [44], GraphCDR [35], SubCDR, and NIHGCN. These models encompass a diverse range of architectures and design principles, including feedforward neural networks, GCN, substructure-aware learning (*e.g.*, molecular fragments or gene sets), and contrastive learning. They utilize various input types, including drug fingerprints, molecular graphs, and multi-omics data (*e.g.*, gene expression, mutation, methylation, and copy number variation). All baseline implementations adhered to the original parameter settings reported in their respective papers.

### Model Performance

#### Performance Imputing Randomly Masked Values (Test 1)

In Test 1, to assess the model’s capacity to reconstruct missing drug sensitivity profiles when neighboring interaction data within the drug-cell line matrix is present, known drug-cell line associations were randomly split into five equal subsets for 5-fold cross-validation. In each fold, one subset served as the test set while the remaining data were used for training. The main results are summarized in Table 2, where drGT ranks among the top three models across all four datasets, achieving AUROC/AUPR of 94.5%/94.3% on the NCI60 dataset, trailing NIHGCN. Overall, NIHGCN achieved the highest scores, followed by MOFGCN, which ranked in the top two. Our model drGT maintained top-three performance across all eight metrics. Unlike NIHGCN and MOFGCN, which operate as black-box models, drGT offers strong predictive performance and interpretability, leveraging ACs to uncover biologically meaningful drug-gene associations. In contrast to SubCDR, which focuses on interaction scores between abstracted features (*e.g.*, molecular fragments or gene sets), drGT directly identifies key biological entities such as individual genes via attention mechanisms, enabling more biologically grounded and literature-supported interpretations.

From an architectural perspective, GNN-based models (MOFGCN, NIHGCN, GraphCDR, SubCDR, and drGT) outperform the feedforward baseline (DeepDSC). Among these, only MOFGCN and GraphCDR integrate multi-omics features such as mutations, methylation, and copy number variation; the others rely on gene expression. Although MOFGCN incorporates multi-omics data, it does not outperform NIHGCN, indicating the difficulty of integrating omics features. Lastly, comparing GNN layers used within drGT reveals that attention-based architectures yield better results than GCN or MPNN. This performance gap reflects enhanced expressiveness of attention mechanisms, enabling models to prioritize relevant neighbors. Although attention-based models are computationally intensive, their ability to prioritize drug-gene or gene-pathway associations that are consistent with known mechanisms or supported by literature evidence may make them suitable for applications demanding both performance and interpretability.

We also evaluated how the choice of padding values for the DGA matrix ($A_{dg}$) affects model performance. Specifically, we compared two strategies: *zero padding*, where unknown associations are treated as absent (value = 0), and *soft padding*, where they are assigned an intermediate value (0.5). As shown in Appendix B, drGT with *zero padding* consistently outperformed drGT with *soft padding* across all datasets, achieving average improvements of 4.7% in AUROC and 4.4% in AUPR over the *soft padding* approach. These results suggest that explicitly treating unknown links as negative (*i.e.*, absent) leads to more reliable predictions.

#### Performance to Unseen Drugs or Cell Lines (Test 2)

Next, we tested the ability of our model to generalize to unseen drugs. This can be a challenging task as models must predict responses for completely unseen drugs or cell lines without direct training examples. Unlike traditional machine learning scenarios, where test samples share similar feature distributions with the training data, our leave-one-out strategy creates a more realistic yet challenging scenario in which models must rely entirely on learned representations and similarities from other drugs or cell lines to make predictions for novel targets. To evaluate the models’ generalization to unseen drugs or cell lines, we adopted a leave-one-out strategy over the cell linedrug interaction matrix, where either a row (cell line) or a column (drug) was held out for testing. The remaining entries served as the training set. We excluded targets with too few or imbalanced labels, specifically, those with fewer than 10 valid entries or with fewer than 2% of either positive or negative labels [52].

After screening, we obtained 853 of 976 drugs and 57 of 59 cell lines in the NCI60 experiments, 297 of 300 drugs and 915 of 925 cell lines in the GDSC1 experiments, 142 of 153 drugs and 498 of 654 cell lines in the GDSC2 experiments, and 2,434 of 470 drugs and 765 of 807 cell lines in the CTRP experiments. Table 3 presents the average AUROC and AUPRC scores for all models on the GDSC2 dataset under the leave-one-out evaluation setting for drugs or cell lines. Results for the remaining datasets are provided in Appendix C.

drGT ranks among the top three, especially for unseen drugs. In the drug-clear-out setting, it achieves AUROC and AUPRC scores within 0.2% and 6.8% of NIHGCN (the best-performing baseline), respectively, and surpasses MOFGCN (the second-best baseline) by 1.4% in AUROC and 3.3% in AUPRC.

In the cell-clear-out setting, drGT achieves an AUROC only 0.5% lower than the second-best baseline, MOFGCN, and surpasses SubCDR, the second-best in AUPR, by 6.8%. While NIHGCN remains the top performer with a substantial margin (+27.6% in AUROC and +26.7% in AUPR over drGT), our method still ranks among the top three, demonstrating competitive performance. Notably, among interpretable methods, drGT achieves the best overall performance, combining predictive accuracy with biological interpretability.

We hypothesize that this trend may relate to the characteristics of the GDSC2 dataset, particularly in terms of response density. For example, GDSC2 contains 10,113 drug cell response entries, with an average of 66.1 non-zero entries per drug and 15.5 per cell, indicating a higher sparsity on the cell side. This difference presents a greater challenge in generalizing to unseen cell lines. The ability of NIHGCN and drGT to maintain strong performance in such settings suggests the benefit of leveraging similarity, whether through learned interactions (as in NIHGCN) or kernel-based similarity matrices (as in drGT).

### Interpretation

We extracted the ACs from multiple GT layers. Each layer includes 5 attention heads. For ease of use, we simplified the shape of the attention matrices from (n, n, 5) to (n, n) by averaging them. We then added these matrices together and used the resulting matrix in subsequent steps.

#### Evaluation of Drug-Gene Associations in Scientific Literature

**Fig. 2.**
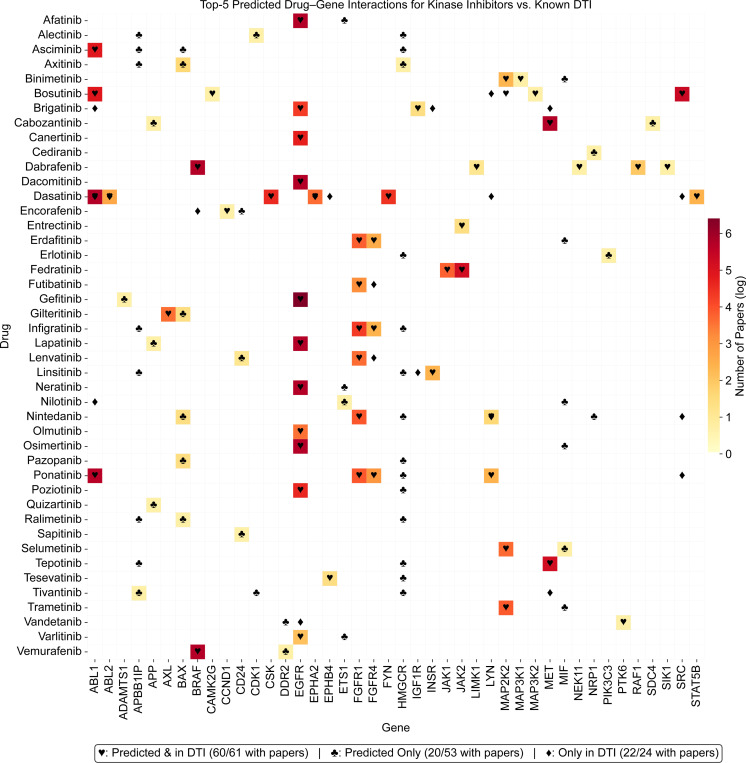
Selected drug-gene co-occurrences based on PubMed abstracts. The color represents the number of abstracts associated with a specific drug and gene pair by natural log scale. Symbols indicate the following: ♥ represents relationships predicted by drGT and present in the DTI dataset (61 instances); ♣ represents relationships predicted by drGT but not present in the DTI dataset (53 instances); ♦ represents relationships only present in the DTI dataset (24 instances). Several drugs have 5 or more co-occurrences due to multiple NSCs. This figure shows a subset of the data for clarity.

We analyzed drug-gene co-occurrences in comparison to PubMed abstracts using the top 5 genes for each drug by AC magnitude. We utilized the NCBI ESearch to retrieve abstracts that mention the drug and gene of each relationship [12]. For this analysis, we focused on the NCI60 dataset, which includes annotated MoA for drugs. To ensure reproducibility and minimize variance due to stochastic training effects, we used the AC matrix from a single fold of Test 1. This yielded two AC matrices—one from the training set and one from the validation set. For downstream analysis, we used the validation AC matrix, as it is unaffected by dropout and thus provides more stable attention patterns. Note that while strict *zero padding* improved predictive performance, we used *soft padding* (0.5) for interpretability analysis. In preliminary experiments, we observed that in the zero-padded setting, ACs were concentrated on known drug-gene links, limiting the model’s ability to identify novel or indirect associations. In contrast, *soft padding* allowed attention to diffuse more broadly, enabling clearer attribution to genes beyond the training annotations. Therefore, ACs analyzed in this section were derived from models trained with *A*_*dg*_ entries for unknown links set to 0.5. Figure 2 displays a heatmap that illustrates the drug-gene co-occurrences based on abstracts. Out of 4,880 drug-gene attention-based relationships, 356 of the drug-gene co-occurrences were found in the abstracts from 16,831 articles. We present this as a subset heatmap in Figure 2, which includes only drugs mentioned by name in at least one abstract, explicitly focusing on compounds whose names end with “-nib”. For both heatmaps, the color corresponds to the number of publications identified by natural log scale. Likewise, the symbols in Figure 2 indicate different relationship types. The ♥ symbol represents relationships predicted by drGT and present in the DTI dataset (61 instances in the subset). The ♣ symbol indicates relationships predicted by drGT but not present in the DTI dataset (53 instances in the subset). The ♦ symbol denotes relationships only present in the DTI dataset (24 instances in subset). Table 4 summarizes the full attention-derived drug-gene associations supported by literature. Across 976 drugs, we extracted 4,880 associations corresponding to the top 5 genes per drug based on ACs. Among these, 4,669 were novel (*i.e.*, not present in the known DTI dataset, while 211 exist in the known DTI dataset (Table 4); this is 36.9 % DTIs of the 572 DTIs included (Table 1). Of all associations, 356 had at least one supporting PubMed abstract. Among the supported associations, 182 were novel, indicating that drGT can identify literature-backed targets that are not present in current DTI datasets. Additionally, 12.2% of novel drugs had at least one PubMed-supported gene among their top-ranked predictions, highlighting the potential of our approach to identify targets for understudied compounds. These results demonstrate that ACs can recover known drug-gene associations and identify under-characterized pairs with supporting literature evidence, suggesting their potential value for further downstream experimental validation.

#### Drug-Target Interactions Assessment from Attention Coefficients

**Fig. 3.**
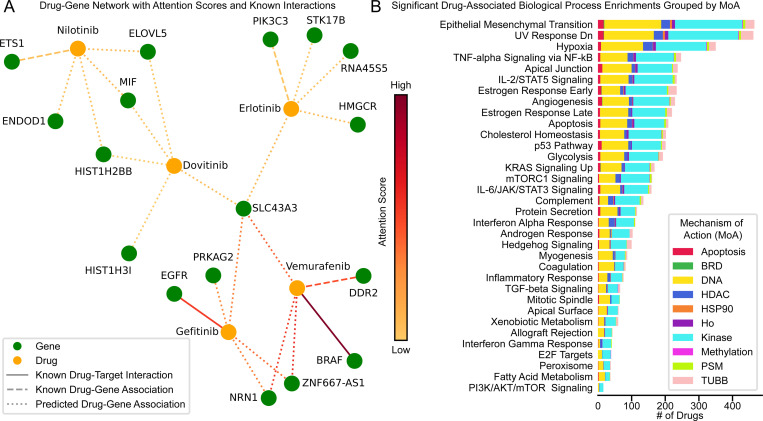
Drug-gene association assessment. (A) DGA network based on ACs. The network shows relationships between drugs (orange nodes) and genes (green nodes) based on the top 5 ACs from drGT. Edges are colored according to the AC value. Lines: known DTIs (*i.e.*, input training data; solid); known DGA (relationships described in abstracts, but not included as model input, dashed); predicted DGAs (dotted). (B) Drug-associated biological processes from gene ACs. A bar chart representing the number of drugs linked to various biological processes, determined by over-representation analysis of hallmark gene sets. X-axis: represents biological processes. Y-axis: number of drugs. The color coding of these bars corresponds to the MoA, including categories such as DNA-targeting agents, tubulin inhibitors (TUBB), kinase inhibitors, hormone-related drugs (Ho), apoptosis inducers, methylation modulators, HDAC inhibitors, HSP90 inhibitors, proteasome inhibitors (PSM), and bromodomain inhibitors (BRD)

The intricate network of DTIs and quantifying their relationships from the model results through ACs aids our further understanding of MoAs. Figure 3A presents a visualization of these DGAs, employing the drGT model to highlight the interplay between drugs and the genes they associate with, focusing on five kinase inhibitors: dovitinib (NSC-759661), erlotinib (718781), gefitinib (759856), nilotinib (747599), and vemurafenib (761431).

Of the kinase inhibitors shown in Figure 3A, gefitinib and vemurafenib are shown with links to their well-known protein targets (EGFR with L858R and BRAF with V600E mutations, respectively) [53, 6].

Within Figure 3, of the drugs with known or plausible drug-gene associations (based on co-occurrences in PubMed abstract text), we observed vemurafenib, erlotinib, and nilotinib. For vemurafenib, our model identified DDR2 (a receptor tyrosine kinase) as a predicted target. In direct validation of our vemurafenib-DDR2 observation, vemurafenib has been shown to decrease the activity of DDR2 to less than 30% [23]. With respect to erlotinib, prior research suggests that inhibition of PIK3C3 (also known as VPS34) can sensitize certain breast cancer cell lines to erlotinib implying that the model may be capturing biologically relevant downstream or compensatory processes [18]. Similarly, for nilotinib, which targets ABL1 and KIT [5], the model identified ETS1, a gene associated with the BCR-ABL signaling pathway [56]. This relates to reports that nilotinib suppresses ETS1 expression [54], suggesting that the model may captures signaling effects that may contribute to the drug’s effect.

In a third category of observations, from the drGT predictions, are recurrent genes that have undergone less direct investigation with individual drugs, but may be of interest for future exploration. Two such genes are NRN1 (identified for gefitinib and vemurafenib) and SLC43A3 (identified for dovitinib, erlotinib, gefitinib, and vemurafenib). NRN1 is a neurotrophic protein (a subset of growth factors) that has been shown to have both tumor suppressive and oncogenic activities related to kinases [17]. NRN1 suppresses esophageal cancer growth by inhibiting PI3K-Akt-mTOR signaling [17], while NRN1 was shown to have a role in oncogenic STAT3 signaling in melanoma [15]. Recent work observed worse prognoses in patients with high SLC43A3 expression. These patients with high SLC43A3 expression also showed enrichment of the PI3K-AKT signalling pathway and enhanced EGF signaling which may relate to erlotinib and gefitinib activity (both EGFR inhibitors) [60]. Dovitinib, a known FGFR family inhibitor [3], was associated with five genes: SLC43A3, MIF, ELOVL5, HIST1H2BB, and HIST1H3I. While these associations have not yet been experimentally explored, they may represent off-target effects or alternative pathways involved in the drug’s MoA—offering novel hypotheses for further investigation.

In summary, the model identifies a set of underexplored genes that may contribute to therapeutic response or resistance across multiple kinase inhibitors. The recurrence of specific genes, such as NRN1, ZNF667-AS1, and SLC43A3, may suggest underlying commonalities.

While not a comprehensive analysis, the above results demonstrate that our model retains high accuracy in classifying known DTIs fed as training data, and also predicts several known drug-gene relationships discussed in the literature. This suggests that our predicted DGAs can be indicative of potential targets or drug-gene relationships that are suitable for study.

#### Over-Representation Analysis with Attention Coefficients

We elucidated the functional roles of genes associated with the various drugs, leveraging the results provided by the ACs derived from our model. We conducted an over-representation analysis (ORA) using the ACs attributed to the genes associated with the drugs. For each drug, the top 100 genes with the highest ACs were analyzed using the gseapy package [19] and the MSigDB Hallmark 2020 [33] collection of 50 gene sets accessed via gseapy. We highlight the biological processes with p-values adjusted by Benjamini-Hochberg less than 0.05 in Figure 3B, showing the drug counts associated with each process. Figure 3B presents the number of drugs related to each enriched biological process, grouped by their corresponding MoA categories. Among the 976 drugs analyzed, 768 exhibited enrichment in at least one biological process (FDR < 0.05). These 768 drugs were categorized as follows: kinase inhibitors (*n* = 322), DNA-targeting agents (*n* = 258), HDAC inhibitors (*n* = 44), tubulin-targeting agents (*n* = 40), apoptosis inducers (*n* = 25), hormone-related agents (*n* = 13), HSP90 inhibitors (*n* = 12), proteasome inhibitors (*n* = 11), methylation modulators (*n* = 4), bromodomain inhibitors (*n* = 3), and agents with other or undefined mechanisms (*n* = 36).

Among these categories, kinase inhibitors showed enrichment for a broad spectrum of biological processes, including epithelial-mesenchymal transition (EMT), KRAS signaling, and IL-2/STAT5 signaling, consistent with their known related pathway [11, 16, 4]. DNA damage drugs were enriched in UV response down, p53 signaling, apoptosis pathways, and xenobiotic metabolism. These findings align with studies linking DNA damage to transcriptional changes (UV response), p53-mediated cell cycle arrest, and induction of apoptosis [25, 1, 22]. Similarly, the involvement of xenobiotic metabolism pathways highlights how these drugs are processed. Other MoAs showed expected associations: HDAC inhibitors with apoptosis [48], hormone-related drugs with apoptosis [31], and tubulin-targeting agents with EMT [57] and UV inhibition of microtubules assembly [63]. Together, these observations highlight how ACs can capture biologically meaningful and MoA-consistent transcriptional programs.

### AC-Derived Targets Align with External Predictors Without Explicit DTI Supervision

To assess the external validity of the DGAs suggested by our model’s ACs, we analyzed the top-5 ranked targets per drug and categorized them based on known DTIs, PubMed co-occurrence, and predictions from DeepPurpose [24], a SOTA DTI prediction model pre-trained on BindingDB [34]. Of the initial 4,880 drug-gene pairs, 4,037 were retained after excluding entries lacking valid SMILES strings or UniProt IDs required by DeepPurpose.

**Fig. 4.**
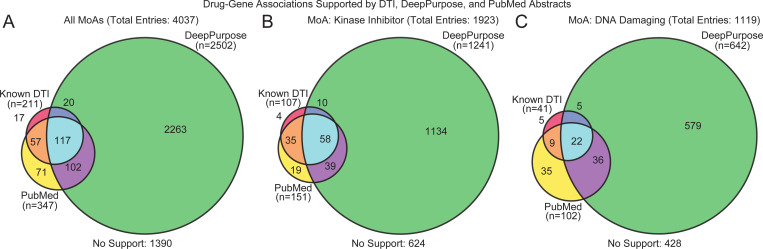
The overlap of DGAs predicted by DeepPurpose, supported by PubMed literature, and known DTI databases (DrugBank). (A) All predicted DGAs (n=4,037). (B) Subset of DGAs targeting kinases (n=1,923). (C) Subset of DGAs targeting DNA (n=1,119). Each circle represents support from one of three sources: known DTIs, DeepPurpose predictions, or PubMed evidence. The number of DGAs supported by each combination is indicated. Entries without support from any of the three sources are listed as “No Support”. All results are in the Appendix E.

Figure 4A summarizes the overlap of evidence support from the three sources: known DTIs, DeepPurpose predictions, and PubMed co-occurrence. Among the 4,037 evaluated DGAs, 2,647 (65.57%) were supported by at least one external source. Specifically, 2,436 out of 3,826 (63.67%) DGAs are supported by PubMed and/or DeepPurpose (the sum of 71, 102, and 2263 entries) even for novel predictions not present in known DTI databases, suggesting our model can recover plausible DTIs even without explicit supervision.

There were 102 entries among the novel predicted DGAs (not present in DTI dataset) and supported by both DeepPurpose and PubMed. These represent the higher-confidence candidates for experimental validation, as they combine computational predictions with literature co-occurrences. The 71 predictions supported by PubMed co-mentions may suggest an association not yet cataloged in DTI databases. Conversely, the substantial number of DeepPurpose-only predictions (2,263 entries) represents predicted but literature-unsupported interactions that may require further investigation. Figure 4B and C show validation by mechanism: 65.64% (1,192/1,816) of kinase inhibitor predictions had external support, versus 60.3% (650/1,078) for DNA-targeting drugs. This likely reflects the better annotation and characterization of kinases. Their structural features also facilitate more accurate predictions. Importantly, across all MoAs, 34.43% of predictions had no external support, but may still represent novel DGAs that require experimental validation.

## Discussion

We developed the drGT model to derive attention coefficients (ACs) for each node, including genes, drugs, and cell lines. This approach is capable of binary drug response prediction and facilitates model interpretation. Furthermore, our model achieves competitive or SOTA performance in both randomly masked and unseen drug/cell settings. Our model provides new connections between drugs and genes, going beyond the original training examples of DTIs. For drug-gene relationships that are not DTIs, our analysis identified potentially novel associations using our AC-based interpretability approach; an approach not found in the other SOTA models compared here. This capability allows our model to not only produce accurate response predictions that utilize known DTI information but also identify potential novel drug-gene relationships. These relationships can be of further use in understanding drug mechanisms. Additionally, we analyzed ACs to understand the processes relevant to each drug.

A limitation of this study, which is also shared by other benchmarked methods, is the lack of a clear mechanistic description of how genes influence drug response. This level of uncertainty also exists in many biological studies that provide explanations that compress dimensions of magnitude, direction, and mechanism in how entities interact, but it is common. Databases, such as the Comparative Toxicogenomics Database, include in their data collections such interactions as they can be the basis of more detailed study [14]. Another challenge in this study is the amount of high-confidence DTI data available. For example, although the NCI60 dataset includes 976 compounds, only 191 drug compounds have DrugBank DTI. This highlights the need for further collection of drug-gene relationships that we have. Ongoing efforts are aimed at expanding such datasets. This expansion utilizes advancements in information extraction with natural language processing as well as crowd-sourced curation [59, 47]. Overall, this work highlights how results from ML techniques can be paired with bioinformatic analyses to understand mechanisms of cellular response to treatment. We expect future work to broaden the approach while providing more granularity to its interpretability. Currently, our model leverages drug response data to generate cell line similarity. Future work could incorporate additional multi-omics data, including methylation, copy number variation, and mutation data, as has been done by others [44]. We believe this enhancement will both boost model performance and interpretability.

## Figures and Tables

**Fig. 1.**
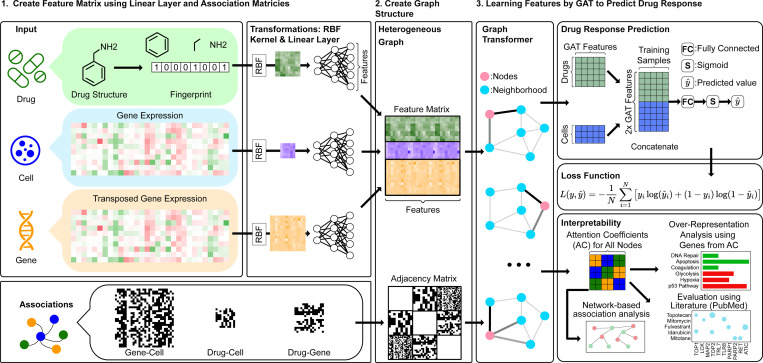
drGT overview. A heterogeneous graph is constructed using drug structures and gene expression data. Each input type is transformed using an RBF kernel, followed by linear layers to project features into a common space. These transformed inputs are then combined into a unified feature matrix, which serves as input to the GT layers along with an adjacency matrix. For prediction, the GT layer output is concatenated to align with the drug response data. Following fully connected (FC) layers, a sigmoid function generates predicted values $\hat{y}$, which are then inputs for the binary cross-entropy loss. To assess interpretability, ACs are utilized in conjunction with over-representation analysis and network-based association analysis.

**Table 1.** Summary statistics of the datasets after preprocessing. The table reports the number of drugs, cell lines, genes, and drug-response pairs, as well as the number of known DTIs. ‘DTI: Drugs and ‘DTI: Genes’ indicate the number of unique drugs and genes involved in at least one DTI.

Counts	NCI60	GDSC1	GDSC2	CTRP
Drugs	976	300	153	470
Cell Lines	59	925	654	807
Genes	2,489	2,106	2,040	2,160
Response Pairs	14,312	31,084	10,113	46,313
Sensitive	7,050	15,149	5,247	24,109
Resistant	7,262	15,935	4,866	22,204
Avg. Observations Per Cell Line	14.66	103.61	66.09	98.54
Avg. Observations Per Drug	242.58	33.60	15.46	57.40

Known DTIs	572	722	313	783
DTI: Drugs	191	82	49	110
DTI: Genes	242	178	100	214

**Table 2.** Performance under 5-fold cross-validation with randomly masked associations (Test 1). Each cell shows the mean (top) and standard deviation (bottom). “Interp.” indicates model interpretability, defined as the ability to reveal biologically meaningful factors contributing to drug response, such as key genes or subcomponent interactions, via mechanisms like attention weights or interaction scores.

	Method	Interp.	NCI60		CTRP		GDSC1		GDSC2	
			AUROC (↑)	AUPR (↑)	AUROC (↑)	AUPR (↑)	AUROC (↑)	AUPR (↑)	AUROC (↑)	AUPR (↑)
Baseline	DeepDSC	**✗**	0.488 (±0.015)	0.490 (±0.014)	0.581 (±0.005)	0.594 (±0.007)	0.591 (±0.006)	0.556 (±0.007)	0.651 (±0.013)	0.648 (±0.015)
	GraphCDR	**✗**	0.924 (±0.006)	0.912 (±0.013)	0.920 (±0.004)	0.931 (±0.005)	0.809 (±0.050)	0.802 (±0.051)	0.871 (±0.011)	0.883 (±0.012)
	SubCDR	**✓**	0.924 (±0.004)	0.909 (±0.010)	0.873 (±0.022)	0.880 (±0.020)	0.750 (±0.046)	0.743 (±0.046)	0.780 (±0.024)	0.782 (±0.026)
	MOFGCN	**✗**	0.956 (±0.003)	0.952 (±0.004)	0.951 (±0.003)	0.956 (±0.003)	0.897 (±0.002)	0.902 (±0.004)	0.908 (±0.007)	0.920 (±0.009)
	NIHGCN	**✗**	**0.965** (±0.002)	**0.963** (±0.002)	**0.956** (±0.002)	**0.960** (±0.002)	**0.914** (±0.003)	**0.915** (±0.004)	**0.928** (±0.009)	**0.935** (±0.011)

drGT	GCN	**✗**	0.633 (±0.011)	0.621 (±0.011)	0.582 (±0.010)	0.588 (±0.008)	0.595 (±0.010)	0.568 (±0.008)	0.648 (±0.010)	0.632 (±0.006)
	MPNN	**✗**	0.538 (±0.031)	0.525 (±0.032)	0.563 (±0.011)	0.567 (±0.008)	0.588 (±0.010)	0.557 (±0.010)	0.641 (±0.014)	0.626 (±0.012)
	GAT	**✓**	0.948^†^ (±0.003)	0.946 (±0.004)	0.924 (±0.002)	0.933 (±0.003)	0.867 (±0.007)	0.869 (±0.007)	0.885 (±0.006)	0.889 (±0.013)
	GATv2	**✓**	0.948^†^ (±0.003)	0.947^†^ (±0.003)	0.927 (±0.002)	0.936 (±0.003)	0.866 (±0.006)	0.867 (±0.006)	0.892 (±0.010)	0.895 (±0.011)
	**Graph Transformer**	**✓**	0.945 (±0.003)	0.943 (±0.004)	0.935^†^ (±0.003)	0.942^†^ (±0.002)	0.884^†^ (±0.002)	0.885^†^ (±0.004)	0.896^†^ (±0.01)	0.903^†^ (±0.01)

**Graph Transformer (our final model)** is in bold. Column bests: **bold** (1st), underlined (2nd), †(3rd).

**Table 3.** Leave-one-out performance on unseen drugs or cell lines (GDSC2).

		Drug		Cell line	
	Methods	AUROC (↑)	AUPR (↑)	AUROC (↑)	AUPR (↑)
Baseline	DeepDSC	0.668 (±0.037)	0.638 (±0.025)	0.693 (±0.296)	0.577 (±0.395)
	GraphCDR	0.588 (±0.289)	0.542 (±0.075)	0.346 (±0.294)	0.499 (±0.137)
	SubCDR	0.728 (±0.082)	0.684 (±0.312)	0.653 (±0.264)	0.634 (±0.346)
	MOFGCN	0.830 (±0.149)	0.793 (±0.205)	0.711 (±0.262)	0.592 (±0.302)
	NIHGCN	**0.846** (±0.181)	**0.894** (±0.164)	**0.982** (±0.025)	**0.969** (±0.043)

drGT	GCN	0.817 (±0.144)	0.800 (±0.183)	0.520 (±0.221)	0.574 (±0.292)
	MPNN	0.789 (±0.161)	0.773 (±0.205)	0.524 (±0.225)	0.578 (±0.296)
	GAT	0.841 (±0.140)	0.825 (±0.177)	0.690 (±0.199)	0.691^†^ (±0.271)
	GATv2	0.843^†^ (±0.139)	0.827 (±0.177)	0.621 (±0.216)	0.634 (±0.280)
	**Graph Transformer**	0.844 (±0.139)	0.826^†^ (±0.180)	0.706^†^ (±0.198)	0.702 (±0.268)

**Table 4.** Validation of Top-5 attention-derived drug-gene associations using PubMed co-occurrence evidence

Metric	Value
Total Predicted Drug-Gene Pairs	4,880
Known (in DTI)	211
Novel (not in DTI)	4,669

Total Predicted Pairs with PubMed Co-Occurrences	356
Novel Pairs with PubMed Support	182
Known Pairs with PubMed Support	174

Drugs with ≥ 1 PubMed-Supported	
Top-5 Novel Pair	12.2%

## Data Availability

Code and data are available https://github.com/sciluna/drGT.
